# Multi-omic characterization of early-onset esophagogastric cancer

**DOI:** 10.1038/s41698-025-01030-4

**Published:** 2025-07-17

**Authors:** Lawrence W. Wu, Sachin Kumar Deshmukh, Sharon Wu, Joanne Xiu, Sung Joo Jang, Jimyung Park, Vincent K. Lam, Emil Lou, Sanjay Goel, Rachna T. Shroff, Ryan H. Moy

**Affiliations:** 1 Division of Hematology/Oncology, Columbia University Irving Medical Center, New York, NY USA; 2 Caris Life Sciences, Phoenix, AZ USA; 3 Division of Surgical Sciences, Columbia University Irving Medical Center, New York, NY USA; 4 Johns Hopkins Sidney Kimmel Comprehensive Cancer Center, Baltimore, MD USA; 5 Division of Hematology, Oncology and Transplantation, University of Minnesota, Minneapolis, MN USA; 6 Rutgers Cancer Institute of New Jersey, New Brunswick, NJ USA; 7 Division of Hematology and Oncology, University of Arizona Cancer Center, Tucson, AZ USA

**Keywords:** Gastric cancer, Oesophageal cancer

## Abstract

Using a large real-world database with matched genomic and transcriptomic data, we characterized clinical and molecular differences between patients with early-onset esophagogastric cancer (EOEGC; <50 years), intermediate-onset esophagogastric cancer (IOEGC; 50-65 years), and average-onset esophagogastric cancer (AOEGC; >65 years). We analyzed clinicopathologic, whole transcriptome, and DNA-sequencing data from 5175 patient samples (EOEGC, *n* = 530; IOEGC, *n* = 1744; AOEGC, *n* = 2901) from the Caris Life Sciences database. Immune deconvolution was performed with quanTIseq and pathway enrichment with Gene Set Enrichment Analysis (GSEA). Real-world overall survival was estimated from insurance claims data. Prevalence of EOEGC was higher in patients who were Black, Asian, Hispanic/Latino, and female. Patients with EOEGC had higher proportion of *CDH1* mutations; *FGFR2*, *CCNE1*, *MYC* copy number alterations; and *ARHGAP26* fusions. Patients with EOEGC had decreased prevalence of immune-oncology markers of microsatellite instability-high, tumor mutation burden-high, and PD-L1 positivity. Immune microenvironment analysis identified significant enrichment of M2 macrophages and decreased M1 macrophages in patients with EOEGC. GSEA identified enrichment of epithelial mesenchymal transition and coagulation pathways in patients with EOEGC. This large real-world characterization of age-stratified esophagogastric cancer found that EOEGC was associated with significant racial, ethnic, and gender differences, and notable molecular differences that may have prognostic and therapeutic implications.

## Introduction

Esophagogastric cancer (EGC) is a highly prevalent cancer globally with over 1.5 million new diagnoses each year^1,2^. The average age of onset for EGC in the United States is 68 years of age. While there has been a decline in EGC rates overall, the incidence of early-onset esophagogastric cancer (EOEGC), defined as age of initial diagnosis less than 50 years old, has strikingly increased over 30% in recent decades^3–8^. Additional studies are needed to understand the reasons for this trend in early-onset disease and to better characterize EOEGC as a disease entity from a molecular perspective.

Known clinical risk factors for EGC include smoking, alcohol consumption, chronic gastroesophageal reflux, *Helicobacter pylori* infection, and obesity^9,10^. The increased incidence of EOEGC may be related to corresponding rising rates of obesity, gastroesophageal reflux disease, and certain environment exposures in the younger population^5,11,12^. Studies to date analyzing germline testing in EOEGC cohorts have identified pathogenic variants in only approximately 25% of patients^13,14^. A recent large single-center study found that compared to patients with EGC diagnosed at age ≥ 50 years old, tumors from patients with EOEGC were more likely to have the genomically stable molecular phenotype, have diffuse-type histology, and unlikely to be microsatellite-instability-high (MSI-H)^15^. Prior studies examining the molecular alterations in EOEGC have been limited to single center or small multi-institutional cohort analyses. The purpose of this study is to compare the distinct molecular and immune microenvironment characteristics of EOEGC and later onset esophagogastric cancer in a large real-world dataset.

## Results

### Patient clinicopathologic characteristics

The study population was composed of 5175 EGC samples with annotated molecular data. 530 (10.2%) patients with EOEGC had a median age of 43 (range 14–49) [Table 1]. In terms of cancer subtype of esophagogastric cancer, patients with EOEGC were characterized by higher proportion of gastric adenocarcinoma compared to patients with IOEGC and AOEGC (53.4% vs 34.8% vs 33.6% respectively; *p* < 0.05) [Table 1]. In terms of tumor specimen site, patients with EOEGC had a higher portion of metastatic site compared to patients with IOEGC or AOEGC (39.4% vs 34.5% vs 24.3% respectively; *p* < 0.05) [Table 1].

**Table 1:** Patient Clinicopathologic Characteristics

			EOEGC	IOEGC	AOEGC
Count (N)			530	1744	2901
Median Age [range]			43 [14–49]	59 [50–64]	73 [65–>89]
**Sex**	Male		66.0% (350/530)	74.9% (1306/1744)	73.7% (2139/2901)
	Female		34.0% (180/530)	25.1% (438/1744)	26.3% (762/2901)
**Lineage**	Esophagogastric Junction Carcinoma		39.4% (209/530)	50.8% (886/1744)	50.7% (1470/2901)
	Gastric Adenocarcinoma		53.4% (283/530)	34.8% (607/1744)	33.6% (974/2901)
	Esophageal Carcinoma		7.2% (38/530)	14.4% (251/1744)	15.7% (457/2901)
**Histology**	Adenocarcinoma		76.4% (405/530)	74.8% (1304/1744)	75.8% (2198/2901)
	Squamous		4.5% (24/530)	10.8% (188/1744)	12.4% (359/2901)
	Other/mixed		19.1% (101/530)	14.4% (252/1744)	11.8% (344/2901)
**Lauren subtype**	Diffuse		12.8% (68/530)	6.1% (107/1744)	5.3% (153/2901)
	Intestinal		0.6% (3/530)	0.9% (16/1744)	1.4% (42/2901)
	Mixed/unclear		86.6% (459/530)	93% (1621/1744)	93.3% (2706/2901)
**Specimen site**	Primary		60.6% (321/530)	65.5% (1162/1744)	75.7% (2197/2901)
	Metastatic	Liver	4.3% (23/530)	4.2% (74/1744)	3.3% (96/2901)
		Peritoneum/Retroperitoneum	7.5% (40/530)	7.4% (129/1744)	6.7% (195/2901)
		Lymph Node	5.3% (28/530)	4.8% (84/1744)	3.4% (99/2901)
		Other	22.3% (118/530)	16.9% (295/1744)	10.8% (314/2901)
**Smoking History**	Yes		94.6% (52/55)	94.4% (303/321)	95.5% (429/449)
	No		5.5% (3/55)	5.6% (18/321)	4.5% (20/449)
**Race**	White		64.2% (174/271)	69.5% (763/1098)	78.8% (2009/2551)
	Black		19.2% (52/271)	16.6% (182/1098)	11.3% (289/2551)
	Asian/Pacific Islander		8.1% (22/271)	6.6% (73/1098)	5.2% (132/2551)
	Other		8.5% (23/271)	7.3% (80/1098)	4.7% (121/2551)
**Ethnicity**	Not Hispanic or Latino		72.5% (195/269)	82.3% (919/1116)	87.9% (2231/2537)
	Hispanic or Latino		27.5% (74/269)	17.7% (197/1116)	12.1% (306/2537)

EOEGC: Age < 50; IOEGC: 50 ≤ Age < 65; AOEGC: Age ≥ 65.

TCGA cohort (Lauren subtype): Diffuse subtype includes signet ring cell carcinoma; Intestinal subtype includes tubular stomach adenocarcinoma, papillary stomach adenocarcinoma, and mucinous stomach adenocarcinoma.

Primary tumor sites were defined as esophagus, gastroesophageal junction, stomach, or gastric. Metastatic tumor sites included liver, peritoneum/retroperitoneum, lymph node, or other (sites not classified as primary or one of the specified metastatic sites).

Race and ethnicity data is self-reported.

The Laurén subtype was limited as most samples were classified as mixed/unclear, but there was a higher proportion of diffuse subtype in patients with EOEGC compared to patients with IOEGC and AOEGC (12.8% vs 6.1% vs 5.3% respectively; *p* < 0.05). While the self-reported history of smoking was limited, in the available data there was no significant difference between patients with EOEGC, IOEGC, and AOEGC (94.6% vs 94.4% vs 95.5% respectively; *p* = 0.757) [Table 1].

Patients with EOEGC had a higher proportion of female gender compared to patients with IOEGC and AOEGC (34.0% vs 25.1% vs 26.3% respectively; *p* < 0.05) [Table 1]. There was a significantly higher proportion of Black race in patients with EOEGC compared to patients with IOEGC and AOEGC (19.2% vs 16.6% vs 11.3% respectively; *p* < 0.05) [Table 1]. Furthermore, there was an increased proportion of Asian race in patients with EOEGC compared to patients with IOEGC and AOEGC (8.1% vs 6.6% vs 5.2% respectively; *p* < 0.05) [Table 1]. Additionally, patients with EOEGC had a higher proportion of Hispanic/Latino ethnicity compared to patients with IOEGC and AOEGC (27.5% vs 17.7% vs 12.1% respectively; *p* < 0.05) [Table 1]. Overall, these data highlight significant disparities related to race, ethnicity, and gender in EOEGC.

### Comparative molecular landscape of EOEGC, IOEGC, and AOEGC

**Fig. 1:**
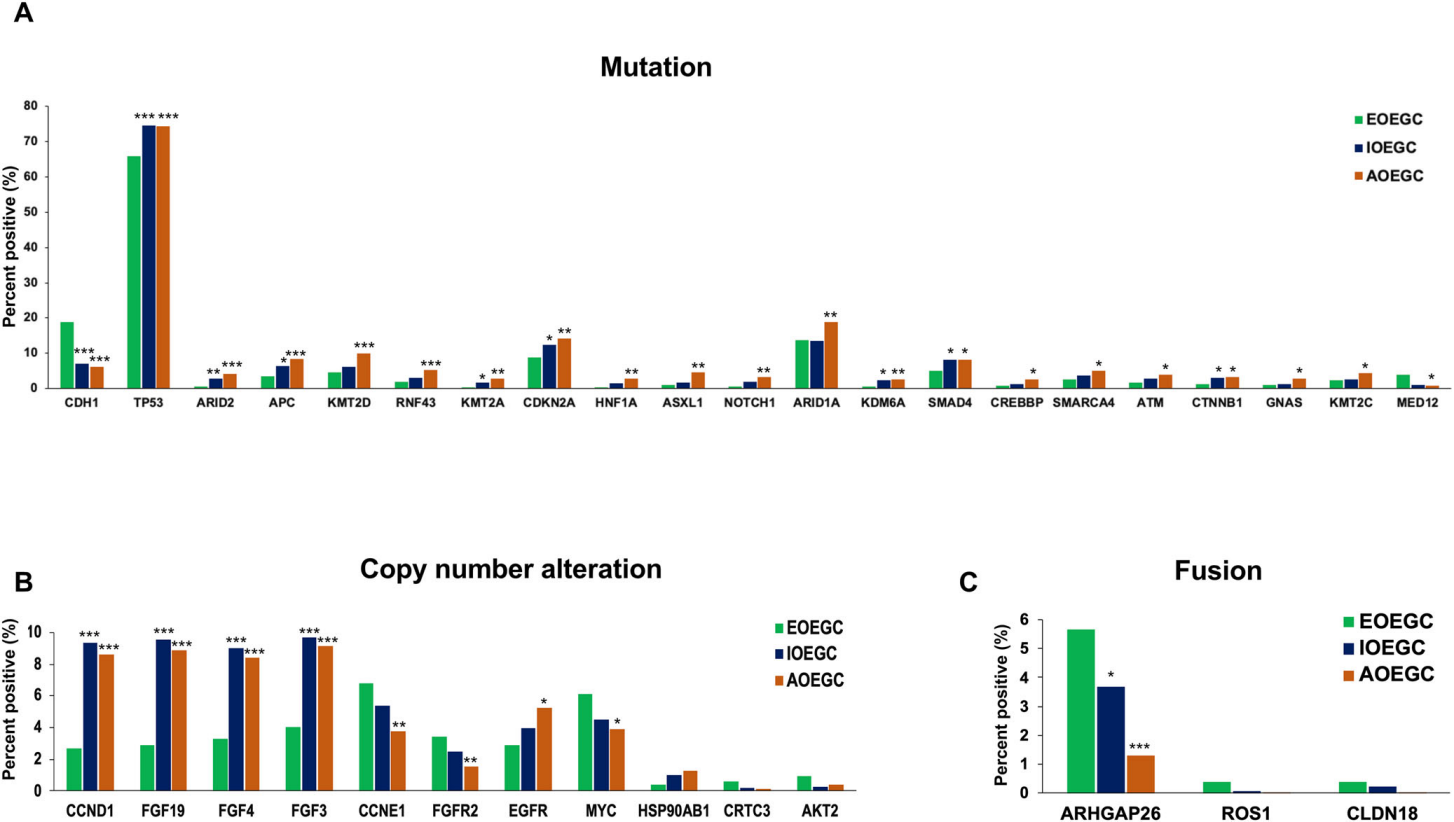
Molecular alterations in EOEGC, IOEGC, and AOEGC. **A** Bar graph of 21 most common mutational alterations. **B** Bar graph of 11 most common copy number alterations. **C** Bar graph of 3 most common fusions. *p < 0.05; **p < 0.01; ***p < 0.001 when compared to EOEGC by chi-square or Fisher’s exact tests.

To determine if EOEGC is associated with distinctive molecular features in a real-world dataset, we analyzed clinically relevant pathogenic/likely pathogenic mutations, copy number alterations, and fusions across the three age cohorts. *TP53* mutations were the most prevalent somatic alterations in patients with EOEGC, IOEGC, and AOEGC. Fewer patients with EOEGC had *TP53* mutations compared to patients with IOEGC (65.9% vs 74.5%; *p* < 0.001, q = 0.006) and AOEGC (65.9% vs 74.4%; *p* < 0.001, q = 0.004) [Fig. 1A, Supplementary Table 1]. *CDH1* mutations were more frequent in patients with EOEGC compared to patients with IOEGC (18.9% vs 7.2%; *p* < 0.001, q < 0.001) and AOEGC (18.9% vs 6.1%; *p* < 0.001, q < 0.001). The most common copy number alterations in patients with EOEGC compared to AOEGC were in *FGFR2* (3.5% vs 1.5%; *p* = 0.002, q = 0.07), *CCNE1* (6.8% vs 3.8%; *p* = 0.002, q = 0.06), and *MYC* (6.1% vs 3.9%, *p* = 0.02, q = 0.33) [Fig. 1B, Supplementary Table 2]. *ARHGAP26* fusions were more prevalent in patients with EOEGC compared to patients with IOEGC (5.7% vs 3.7%; *p* = 0.04, q = 0.53) and AOEGC (5.7% vs 1.3%; *p* < 0.001, q < 0.001) [Fig. 1C, Supplementary Table 3]. The *ARHGAP26* fusion components are in Supplementary Table 4. There were no major differences between cohorts in HER2 positivity by RNA, IHC, copy number alteration, and chromogenic in situ hybridization, except for increased HER2 positivity by CISH in patients with IOEGC compared to patients with EOEGC [Supplementary Fig. 1, Supplementary Table 5].

**Fig. 2:**
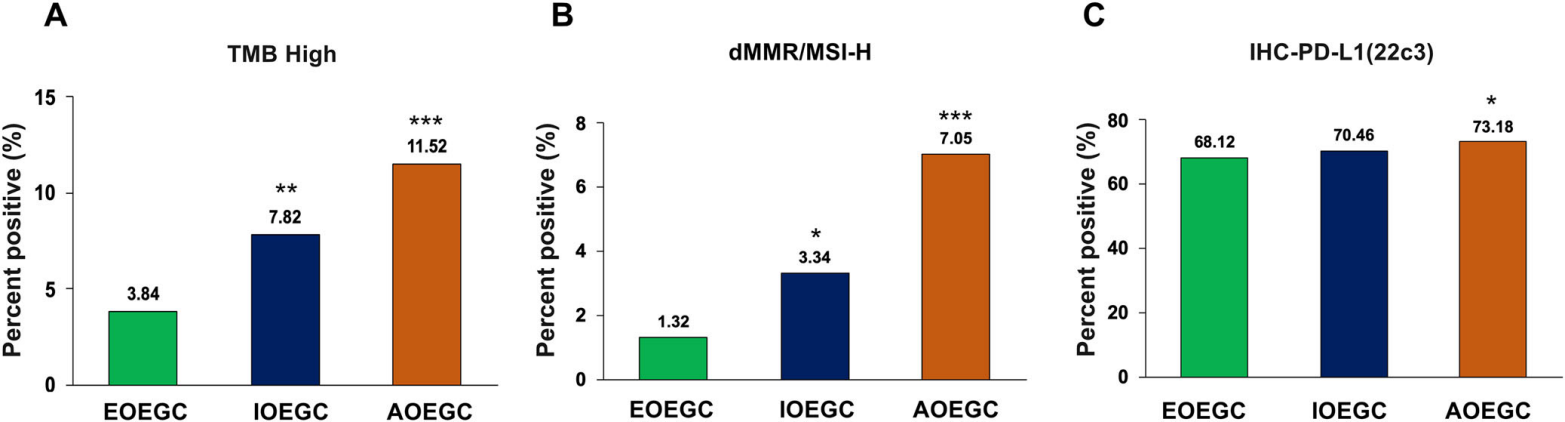
Immuno-oncology biomarkers in EOEGC, IOEGC, and AOEGC. **A** Bar graph for TMB-High status (≥10 mutations/MB). **B** Bar graph for dMMR/MSI-H status. **C** Bar graph for PD-L1 positive status as determined by immunohistochemistry from the 22c3 assay. *p < 0.05; **p < 0.01; *** *p* < 0.001 when compared to EOEGC by chi-square or Fisher’s exact tests.

Patients with EOEGC had decreased positivity of several immune-oncology markers that predict response to immune checkpoint inhibition. Patients with EOEGC had decreased rates of TMB-high compared to patients with IOEGC (3.8% vs 7.8%; *p* = 0.002, q = 0.05) and AOEGC (3.8% vs 11.5%; *p* < 0.001, q < 0.001) [Fig. 2A, Supplementary Table 6]. Patients with EOEGC also had decreased prevalence of dMMR/MSI-H compared to patients with IOEGC (1.3% vs 3.3%; *p* = 0.02, q = 0.28) and AOEGC (1.3% vs 7.1%; *p* < 0.001, q < 0.001) [Fig. 2B, Supplementary Table 6]. Although patients with EOEGC had no difference in PD-L1 positivity compared to patients with IOEGC (68.1% vs 70.5%; *p* = 0.32, q = 1.00), they demonstrated decreased PD-L1 positivity compared to patients with AOEGC (68.1% vs 73.2%; *p* = 0.02, q = 0.34) [Fig. 2C, Supplementary Table 6]. Taken together, these data highlight several notable differences in somatic mutations, copy number alterations, fusions, and common immune-oncology markers in patients with EOEGC.

### Characterization of the immune tumor microenvironment and immune checkpoint gene expression

Given the decreased positivity of immune-oncology biomarkers in patients with EOEGC, we performed further analyses to quantify immune cell infiltration and immune gene expression.

**Fig. 3:**
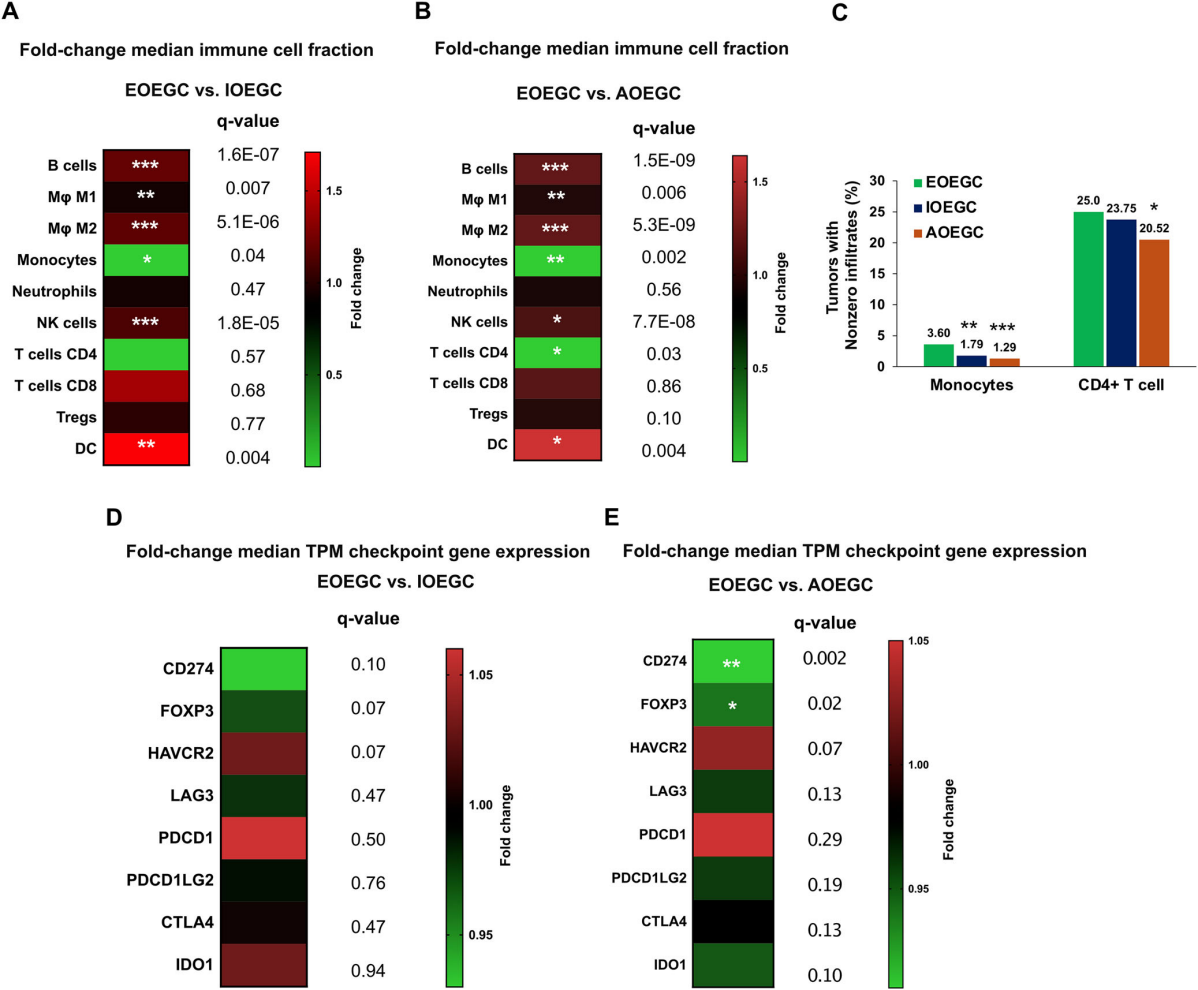
Immune cell infiltrate and immune gene expression in EOEGC, IOEGC, and AOEGC. **A** Computationally inferred intratumoral immune population. The heatmap indicates fold change of IOEGC relative to EOEGC in median immune fraction according to quanTIseq. **B** The heatmap indicates fold change of AOEGC relative to EOEGC in median immune fraction according to quanTIseq. Tumor microenvironment cell fractions were analyzed among cohorts using nonparametric Kruskal-Wallis testing. The Benjamini-Hochberg method was utilized to adjust p-values for multiple comparisons (*q < 0.05; **q < 0.01; ***q < 0.001). **C** For cell types with median values of “0”(Ex: monocytes and CD4^+^ T cells), the percentage of tumors with nonzero immune infiltrates were compared. **D** Fold change gene expression levels in transcripts per million (TPM) of immune checkpoint genes IOEGC relative to EOEGC. **E** Fold change gene expression levels in TPM of immune checkpoint genes AOEGC relative to EOEGC. Mann-Whitney U test used to determine statistically significant differences in immune gene expression. The Benjamini-Hochberg method was utilized to adjust p-values for multiple comparisons (*q < 0.05; **q < 0.01; ***q < 0.001).

There was enrichment of B-cells (*p* < 0.001, q < 0.001), M2 macrophages (*p* < 0.001, q < 0.001), natural killer cells (*p* < 0.001, q < 0.001), dendritic cells (*p* = 0.001, q = 0.004), monocytes (*p* = 0.0004, q = 0.002), and CD4 + T cells (*p* = 0.01, q = 0.03) in tumors from patients with EOEGC compared to patients with AOEGC [Fig. 3A, B, Supplementary Table 7]. In contrast, there was decreased relative enrichment of M1 macrophages (Fold change 0.93; *p* = 0.002, q = 0.006) in tumors from patients with EOEGC compared to patients with AOEGC. A similar trend of immune cell infiltrates was observed when comparing between tumors from patients with EOEGC and IOEGC.

For immune checkpoint gene expression analysis, there was increased *HAVCR2* expression in tumors of patients with EOEGC compared to patients with IOEGC (Fold change 1.03; *p* = 0.02, q = 0.07) and AOEGC (Fold change 1.03; *p* = 0.02, q = 0.07) [Fig. 3D, E, Supplementary Table 8]. Conversely, there was decreased *CD274* and *FOXP3* expression in tumors of patients with EOEGC compared to patients with IOEGC and AOEGC. There were no significant differences in other key immune checkpoint genes such as *LAG3*, *CTLA4*, and *IDO1*.

### Differentially regulated signaling pathways in tumor transcriptomes in EOEGC, IOEGC, and AOEGC

To complement the mutational profiling, immuno-oncology markers, and immune microenvironment analyses in this study, we performed gene set enrichment analysis to identify potential underlying pathways in patients with EOEGC. A positive normalized enrichment score (NES) was indicative of enhanced pathway signalling in the given GSEA gene set.

**Fig. 4:**
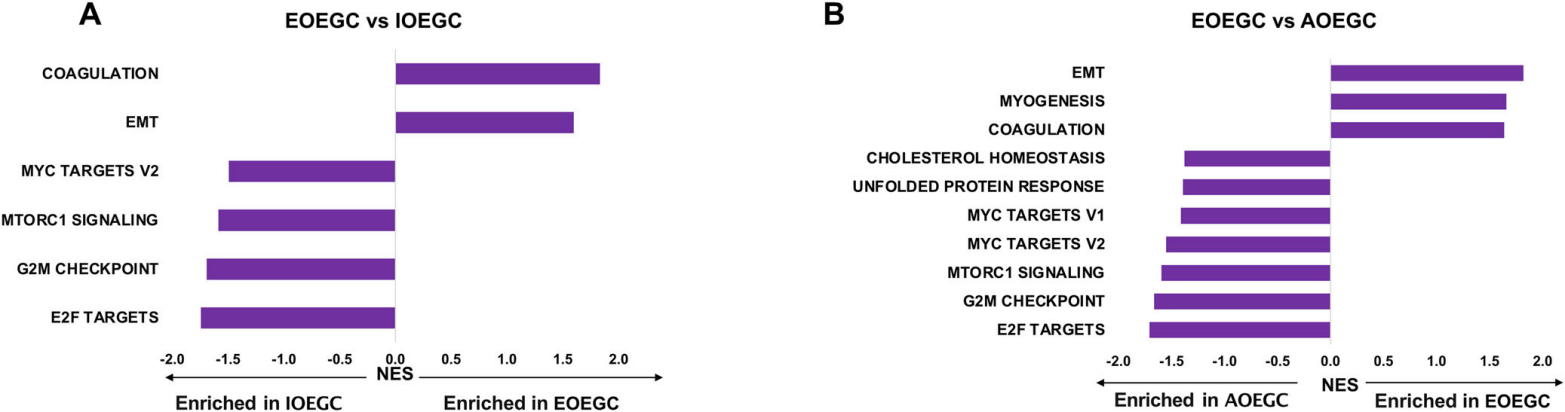
Gene set enrichment analysis (GSEA) in EOEGC, IOEGC, and AOEGC. **A** GSEA differences in pathways based on normalized enrichment scores (NES) in EOEGC versus IOEGC. **B** GSEA differences in pathways based on NES in EOEGC versus AOEGC. Positive NES would imply higher values in EOEGC. All pathways with false discovery rate (FDR) <0.25.

Patients with EOEGC had significant enrichment of epithelial-to-mesenchymal transition (EMT) genes compared to patients with IOEGC (NES = 1.83, false discovery rate (FDR) = 0.00) and AOEGC (NES = 1.82, FDR = 0.00) [Fig. 4, Supplementary Tables 9, 10]. There was also significant enrichment of coagulation genes in patients with EOEGC compared to patients with IOEGC (NES = 1.60, FDR = 0.02) and AOEGC (NES = 1.64, FDR = 0.04). Conversely, there was significant overrepresentation of the genes related to E2F targets, G2M checkpoint pathway, MYC targets, and mTORC1 signaling targets in both patients with IOEGC and AOEGC compared to patients with EOEGC [Fig. 4, Supplementary Table 10].

Additionally, the MAPK pathway activity score was decreased in patients with EOEGC compared to patients with IOEGC (MPAS score 0.04; *p* = 0.04, q = 0.06) and AOEGC (MPAS score 0.18; *p* = 0.001, q = 0.004) [Supplementary Fig. 2, Supplementary Table 11].

### Clinical outcomes of patients with EOEGC, IOEGC, and AOEGC

**Fig. 5:**
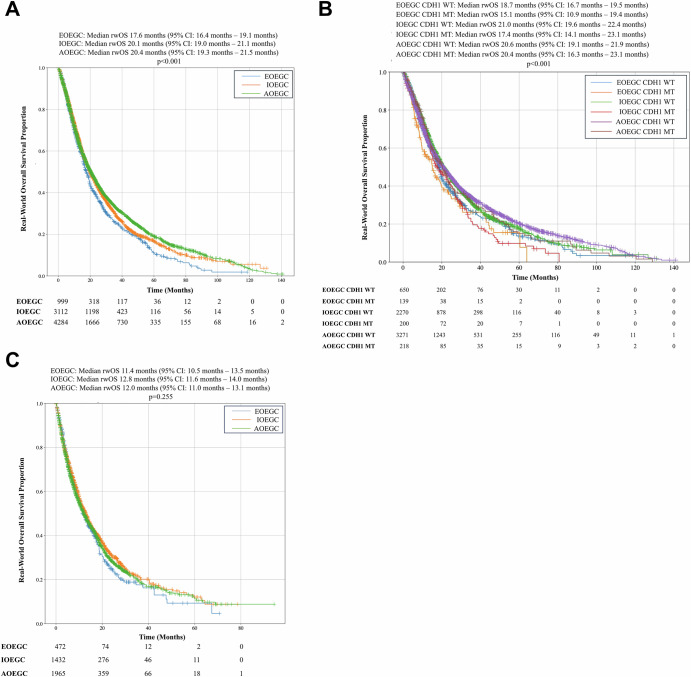
Real world overall survival (rwOS) for patients with EOEGC, IOEGC, and AOEGC stratified by treatment. **A** Kaplan-Meier curves of rwOS of patients with EOEGC, IOEGC, AOEGC after treatment with chemotherapy (Defined as Carboplatin, Cisplatin, Docetaxel, Fluorouracil, Oxaliplatin, Paclitaxel, FOLFIRI, FOLFOX, FOLFIRINOX). **B** rwOS stratified by *CDH1*-mutation status after treatment with chemotherapy. **C** rwOS after treatment with immune checkpoint inhibitor with pembrolizumab or nivolumab. Log-rank test was performed with significance determined as *p* < 0.05.

Real world overall survival (rwOS) was calculated with insurance claims-related follow-up. We observed significantly reduced rwOS after chemotherapy in patients with EOEGC compared to patients with IOEGC and AOEGC (Median rwOS 17.6 months vs 20.1 months vs 20.4 months respectively; *p* < 0.001) [Fig. 5A]. When further stratified by *CDH1* mutation status, patients with EOEGC who were *CDH1*-mutated had the worst survival after chemotherapy of all subgroups, including patients with EOEGC who were *CDH1*-wildtype, patients with AOEGC whose tumors were *CDH1*-mutated, and patients with AOEGC with *CDH1*-wildtype tumors (Median rwOS 15.1 months versus 18.7 months versus 20.4 months versus 20.6 months respectively; *p* < 0.001) [Fig. 5B]. For tumors that were HER2-positive, there was no significant difference in rwOS for patients with EOEGC treated with trastuzumab compared to patients with IOEGC or AOEGC [Supplementary Fig. 3C].

Although biomarkers associated with clinical response to immune checkpoint inhibition, such as TMB-High and dMMR/MSI-H status, were less prevalent in patients with EOEGC, there was no significant difference in rwOS after immune checkpoint inhibitor treatment with pembrolizumab or nivolumab in patients with EOEGC compared to patients with IOEGC and AOEGC (Median rwOS 11.4 months vs 12.8 months vs 12.0 months respectively; p = 0.255) [Fig. 5C]. When stratified by dMMR/MSI-H status, there remained no difference in rwOS in patients after immune checkpoint inhibitor treatment in patients with EOEGC compared to patients with IOEGC and AOEGC (Median rwOS not reached vs 30.9 months vs 21.2 months respectively; *p* = 0.116) [Supplementary Fig. 3A]. Moreover, when stratified by TMB High status, there remained no difference in rwOS in patients after immune checkpoint inhibitor treatment in patients with EOEGC compared to patients with IOEGC and AOEGC (Median rwOS 18.7 months vs 19.8 months vs 16.9 months respectively; *p* = 0.541) [Supplementary Fig. 3B].

## Discussion

To our knowledge, this study is the largest real-world investigation of 5175 patients with esophagogastric cancer and associated comprehensive analyses of molecular and immunologic features. We identified several differences in demographics, molecular characteristics, and immunologic features between EOEGC and older-onset esophagogastric cancer.

The largest single institution study at Memorial Sloan Kettering Cancer Center analyzing clinical and molecular features of EOEGC (*n* = 219) provided useful insight into several differing molecular and clinical characteristics^15^. Our data is consistent with previously observed findings including increased incidence in female patients, increased *CDH1* and *CCNE1* alterations, and decreased prevalence of MSI-H. Our study expands upon existing knowledge by leveraging a large and diverse real-world cohort with highly annotated molecular and transcriptomic data.

Prior studies characterizing early versus average onset cancers utilize age of diagnosis at 50 as a discriminatory cutoff, which may be limiting as the average age of onset for EGC is approximately 68 years old^7,15^. Our study provides additional granularity by stratifying patients with EGC who are were diagnosed between age ≥50 and <65 (IOEGC) and diagnosed at age ≥ 65 years (AOEGC). Our data highlight racial, ethnic, and gender disparities in EOEGC with higher incidence in Black, Asian/Pacific Islander, Hispanic/Latino, and Female populations. We postulate that these disparities may be potentially related to rising incidence of diffuse-type gastric cancer in these populations^16–21^.

Key genomic alterations that were enriched in patients with EOEGC included *CDH1*, *FGFR2*, *CCNE1*, *MYC*, and *ARHGAP26* fusions. *CDH1* alterations are commonly found in diffuse-type gastric cancer and germline mutations are strongly associated with hereditary diffuse gastric cancer syndrome^22,23^. *FGFR2* alterations are relatively uncommon in gastric cancer, but have important clinical implications given the promising efficacy of FGFR2-targeting therapeutics such as bemarituzumab^24,25^. *CCNE1* amplifications have been linked to worse prognosis after treatment with trastuzumab^26,27^. There are ongoing clinical trials utilizing Wee1 kinase inhibitor adavosertib and protein kinase membrane associated tyrosine/threonine 1 (PKMYT1) inhibitor lunresertib to target tumors with *CCNE1* amplifications^28,29^. *MYC* has traditionally been thought to be undruggable, but there are several ongoing preclinical and clinical studies directly and indirectly targeting *MYC*^30^. ARHGAP26 fusions are structural rearrangements that often involve Claudin 18 (CLDN18) and are also enriched in diffuse-type gastric cancer^31,32^. Taken together, there is significant enrichment of molecular alterations associated with diffuse-type gastric cancer and also alterations that may be targetable with emerging treatments being tested in ongoing clinical trials.

In terms of immune-oncology markers, patients with EOEGC had decreased prevalence of dMMR/MSI-H, TMB-High, and PD-L1 positivitity, which are each biomarkers associated with response to immune checkpoint inhibition. To provide further insight into the immune microenvironment, we analyzed immune cell infiltrate and observed increased B-cells, M2 macrophages, natural killer cells, dendritic cells, monocytes, and CD4 + T cells in tumors from patients with EOEGC. There was also decreased M1 macrophages in tumors from patients with EOEGC. Broadly speaking, M1 macrophages are considered pro-inflammatory and M2 macrophages are considered anti-inflammatory^33^. Further analyses in gastric cancer have identified increased M1 macrophages associated with improved prognosis and increased M2 macrophages associated with worse prognosis, diffuse-type histology, and more advanced stage^34^. Additionally in esophagogastric cancer, a lower M1/M2 ratio has been associated with poorer prognosis^35,36^. There have been preclinical efforts to target tumor associated macrophages and induce polarization from M2 macrophages to M1 macrophages^37^. Pexidartinib, a colony-stimulating factor 1 receptor (CSF-1R) inhibitor, has shown synergy with PD-1 inhibition to induce macrophage repolarization towards M1 macrophages in a preclinical model of esophageal adenocarcinoma^38,39^. Another target includes Dickkopf-related protein (DKK1) for which elevated expression is associated with worsened survival in gastric cancer, increased M2 macrophages, and an immunosuppressive microenvironment^40^. DKN-01, a humanized IgG4 monoclonal antibody against circulating DKK1, is able to repolarize macrophage subsets towards M1 macrophages and showed promising clinical results in a phase II trial in combination with tislelizumab (anti-PD-1) and chemotherapy in the first line setting for advanced HER2-negative gastric/gastroesophageal adenocarcinoma^41^. Overall, these data represent a potentially unfavorable tumor immune microenvironment with relative decreased M1 macrophages and increased M2 macrophages in patients with EOEGC and a potential therapeutic opportunity.

GSEA identified genes related to EMT and coagulation to be significantly enriched in patients with EOEGC compared to both IOEGC and AOEGC. EMT is enriched in diffuse-type and plays an important role in its pathogenesis^42–45^. A study analyzing high expression of coagulation genes from the TCGA in gastric cancer identified an association with increased tumoral angiogenesis, increased EMT gene expression, low tumor mutation burden, and worse patient prognosis^46^. These differences in GSEA genes may be potentially related to enrichment of diffuse-type gastric cancer in the early onset group.

Our survival data identified decreased rwOS in patients with EOEGC after chemotherapy compared to patients with IOEGC and AOEGC. This worsened survival was most pronounced in patients with EOEGC with *CDH1*-mutated tumors. This decreased survival may be related to chemotherapy resistance associated to enrichment of EMT, which is frequently seen in patients with *CDH1* mutations^47^. While there was decreased prevalence of key biomarkers related to response to immune checkpoint inhibition such as dMMR/MSI-H and PD-L1 positivity in patients with EOEGC, there was no corresponding decreased rwOS in patients with EOEGC who receive immune checkpoint inhibitors. We hypothesize that patients who received immune checkpoint inhibitors were likely prescribed treatment based on biomarkers, exhibiting tumor characteristics such as dMMR/MSI-H or PD-L1 positivity, and that these biomarkers may have contributed to a favorable response to treatment across cohorts. Nonetheless, since there was decreased prevalence of biomarkers related to ICI response in patients with EOEGC, there were less patients eligible to receive beneficial treatment with ICI.

There are several limitations of our study. The Caris CODEai database lacks clinical annotation of certain clinicopathologic variables such as such as performance status, method of tissue acquisition, and stage of diagnosis and had limited information on the Laurén histological subtype. There was very limited Claudin 18.2 immunohistochemistry testing in this study cohort (<2 samples per age cohort), but this assay has been recently incorporated into standardized profiling for EGC, which will enable assessment in the future. Patients with advanced disease are more likely to undergo molecular testing and may not be representative of the entire population of EGC. Bulk tumor sequencing limits the ability to fully characterize cell-specific molecular features and future studies may utilize single-cell RNA sequencing, spatial transcriptomics, or quantitative immunofluorescence to provide further insight into tumor microenvironment specific interactions and features.

In summary, EOEGC is characterized by racial, ethnic, and gender disparities, increased *CDH1* mutations, decreased prevalence of markers of response to ICI, and enrichment of pathways related to EMT and coagulation. Our data appear largely concordant with other published reports studying EOEGC and adds important insight into immune cell infiltrate and pathway alterations. We hypothesize that the rise of EOEGC may be related to the ongoing rise in diffuse-type gastric cancer given similar trends in mutations, molecular biomarkers, and pathway enrichment. There remains an ongoing need to further elucidate the impact of environment factors on increased EOEGC incidence. Overall these unique differential characteristics present therapeutic opportunities, but also demonstrate the limitations of currently approved therapies in patients with EOEGC.

## Methods

### Patient samples

5175 EGC samples were identified in the Caris Life Sciences database (Phoenix, AZ) with matched DNA sequencing, whole-transcriptome sequencing (WTS), and immunohistochemistry (IHC) data. We defined EGC esophageal squamous cell carcinoma, esophageal adenocarcinoma, gastric adenocarcinoma, and other/mixed histologies occurring in the esophagus, esophagogastric junction, or stomach.

We defined EOEGC as younger than 50 years old at diagnosis (n = 530). While prior studies have defined AOEGC as age ≥ 50 years old, we utilized two age cohorts given the average age of diagnosis of EGC is approximately 68 years old^7,8^. We defined intermediate-onset EGC (IOEGC) as age ≥50 and <65 at diagnosis (*n* = 1744) and AOEGC as age ≥ 65 years at diagnosis (n = 2901). Histology and Laurén subtype were included when available. We classified site of tumor specimen as primary or metastatic. Metastatic tumor sites included liver, peritoneum/retroperitoneum, lymph node, or other (sites not classified as primary or one of the specified metastatic sites). Smoking history and self-reported race and ethnicity were included when provided.

### Next Generation Sequencing (NGS)

NGS was performed on genomic DNA isolated from formalin fixed paraffin-embedded (FFPE) tumor samples using the NextSeq or NovaSeq 6000 platforms (Illumina, Inc., San Diego, CA) as previously described^48^. For NextSeq-sequenced tumors, a custom-designed SureSelect XT assay was used to enrich 592 whole-gene targets (Agilent Technologies, Santa Clara, CA). For NovaSeq-sequenced tumors, a hybrid pull-down panel of baits designed to enrich for >700 clinically relevant genes at high coverage and read depth was used, along with a separate panel to enrich for an additional >20,000 genes at lower depth. Genetic variants were detected with >99% confidence and were categorized by board-certified molecular geneticists. Only mutations previously classified as “pathogenic” and “presumed pathogenic” were included in the mutation frequency analysis.

### Immunohistochemistry (IHC)

IHC was performed using automated staining techniques on slides obtained from FFPE tumor blocks as previously described (Benchmark XT; Ventana; and AutostainerLink 48; Dako, Carpinteria, CA)^49^. PD-L1 (22c3) and HER2 (4B5) was stained and scored according to previous criteria^50^. Staining was scored for intensity (0: negative; 1: weak; 2: moderate; 3: strong) and staining percentage (0–100%). Chromogenic in situ hybridization (CISH) was used for HER2/neu amplification (INFORM HER2 Dual ISH DNA Probe Cocktail)^50^. A HER2-CEP17 ratio of ≥2 was defined as positive for HER2 amplification.

### Tumor Mutational Burden (TMB)

TMB was measured (592 genes and 1.4 megabases [MB] sequenced per tumor) as previously described by counting all non-synonymous missense mutations found per tumor that had not been previously described as germline alterations according to dbSNP and 1KG databases^51^. TMB-High was defined as ≥10 mutations/MB per the KEYNOTE-158 trial^52^.

### Mismatch repair deficiency and microsatellite instability

A combination of multiple test platforms was used to determine the mismatch repair deficiency (dMMR)/MSI-H status of the tumors, including fragment analysis (FA, Promega), IHC [MLH1, M1 antibody; MSH2, G2191129 antibody; MSH6, 44 antibody; and PMS2, EPR3947 antibody (Ventana Medical Systems, Inc.)], and NGS. The three platforms generated highly concordant results as reported previously^53^. In the rare cases of discordant results, dMMR/MSI-H status was determined in the order of IHC, FA, and NGS.

### Whole Transcriptomic Sequencing (WTS)

Tumor-specific mRNA was isolated with a Qiagen RNeasy FFPE Kit (Germantown, MD) from manually microdissected areas of FFPE sections with a tumor content of at least 10%. Quality and quantity were determined with the Agilent TapeStation (Santa Clara, CA). Library preparation and whole-transcriptome sequencing were performed on the NovaSeq 6500 platform (Illumina, Inc, San Diego, CA) to an average of 60 M reads as previously described^48^. Gene fusions were detected using the ArcherDX fusion assay (ArcherDX, Boulder, CO) and Illumina MiSeq platform (Illumina MiSeq, San Diego, CA) as previously described^54^. Gene set enrichment analysis (GSEA) was performed on WTS data^55^. MAPK pathway activation score was calculated using a 10-gene set (*SPRY2*, *SPRY4*, *ETV4*, *ETV5*, *DUSP4*, *DUSP6*, *CCND1*, *EPHA2*, and *EPHA4*)^56^. Immune checkpoint gene expression was analyzed for *CD274*, *FOXP3*, *HAVCR2*, *LAG3*, *PDCD1*, *PDCD1LG2*, *CTLA4*, and *IDO1*. Immune cell infiltrate fractions were calculated from transcriptomic data using quanTIseq^57^.

### Clinical outcomes

Real-world overall survival (rwOS) data was obtained from insurance claims data and calculated from the date of tissue collection or treatment start as a surrogate for diagnosis until date of last contact. Patients without contact/claims data for a period of at least 100 days were presumed deceased. Conversely, patients with a documented clinical activity within 100 days prior to the latest data update were censored in the analysis. Kaplan-Meier estimates were calculated for EOEGC, IOEGC, and AOEGC cohorts within the broader Caris CODEai clinicogenomic database. Hazard ratios (HR) were determined by Cox Proportional Hazards model and rwOS differences were assessed with the log-rank test, with significance defined as *p* < 0.05.

### Statistical analysis

Statistical significance was determined using chi-square, Fisher exact and Mann-Whitney U test as appropriate. The Benjamini-Hochberg method was utilized to adjust p-values for multiple comparisons (q < 0.05). Tumor immune microenvironment cell fractions were analyzed among cohorts using nonparametric Kruskal-Wallis testing. RwOS utilized the log-rank test with significance defined as *p* < 0.05.

### Compliance statement

This study was conducted in accordance with guidelines of the Declaration of Helsinki, Belmont report, and U.S. Common rule. Per 45 CFR 46.101(b), this study was performed utilizing retrospective, deidentified clinical data. Therefore, this study is considered Institutional Review Board exempt and informed consent was not required.

## Supplementary information

EOEGC Supplementary Figures 050425

## Data Availability

The data presented in this study are not publicly available due to data size and patient privacy but are available on reasonable request from the corresponding author. The NGS raw data are owned by Caris Life Sciences and cannot be publicly shared because of the data usage agreement signed by Dr. Ryan H. Moy. Qualified researchers can apply for access to these data by contacting Joanne Xiu (jxiu@carisls.com) and signing a data usage agreement.
