# Supplementary material for: Multi-omic characterization of early-onset esophagogastric cancer
Source: NPJ Precis Oncol. 2025 Jul 17;9:241. doi: 10.1038/s41698-025-01030-4 (PMC12271507; doi:10.1038/s41698-025-01030-4)
Supplement: Supplementary file 1 — EOEGC Supplementary Figures 050425 [file 41698_2025_1030_MOESM1_ESM.pdf]

Supplementary Figure 1: HER2 expression and amplification analyses for EOEGC, IOEGC, AOEGC. (A) RNA expression in transcripts per million (TPM). (B) Immunohistochemistry (IHC). (C) *ERBB2* copy number alterations (CNA). (D) Chromogenic in situ hybridization to determine HER2/neu amplification. Mann-Whitney U test was used to compare cohorts to EOEGC with * p<0.05; ** p<0.01; *** p<0.001.

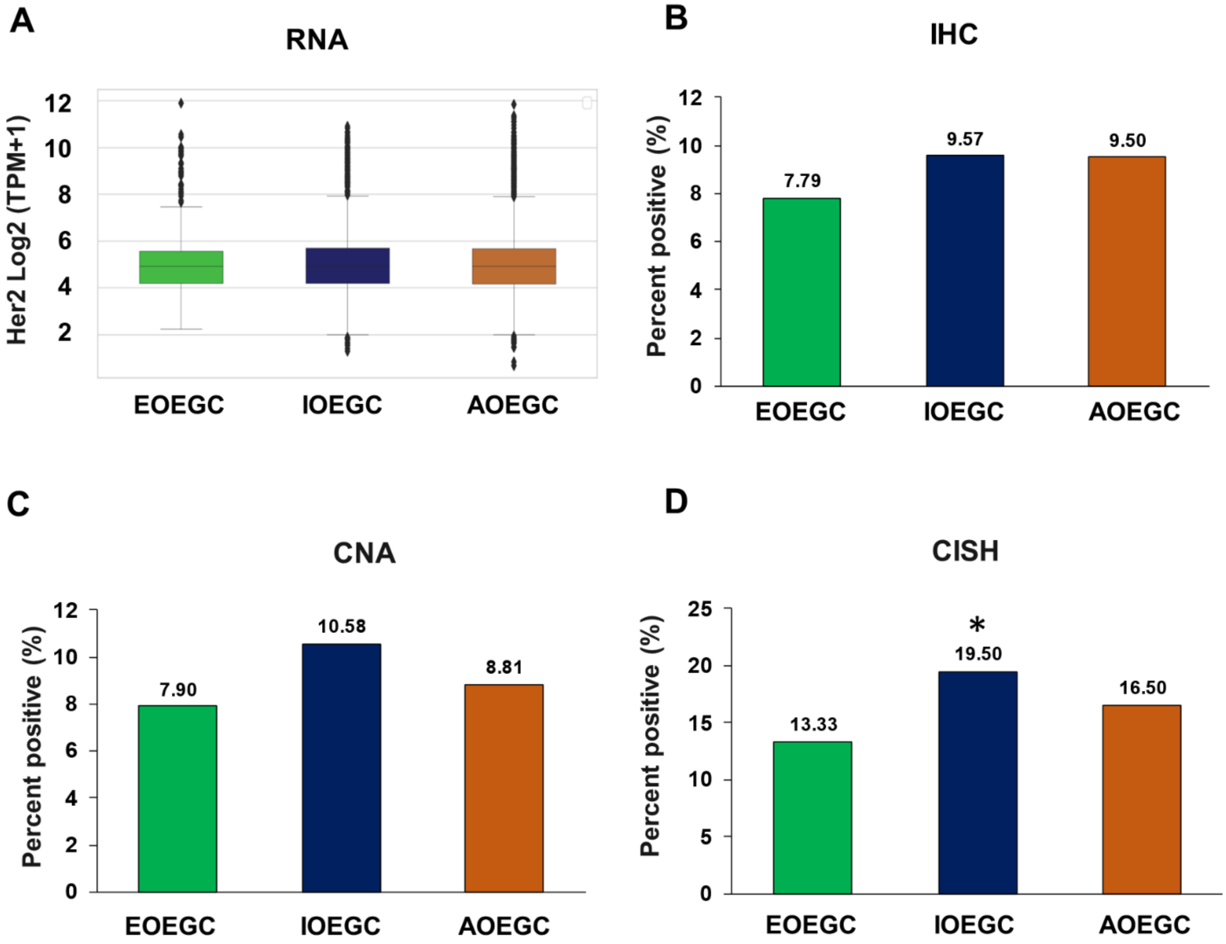

Supplementary Figure 2: MAPK Pathway Activity Score. Mann-Whitney U test was used to compare cohorts to EOEGC with * p<0.05; ** p<0.01; *** p<0.001.

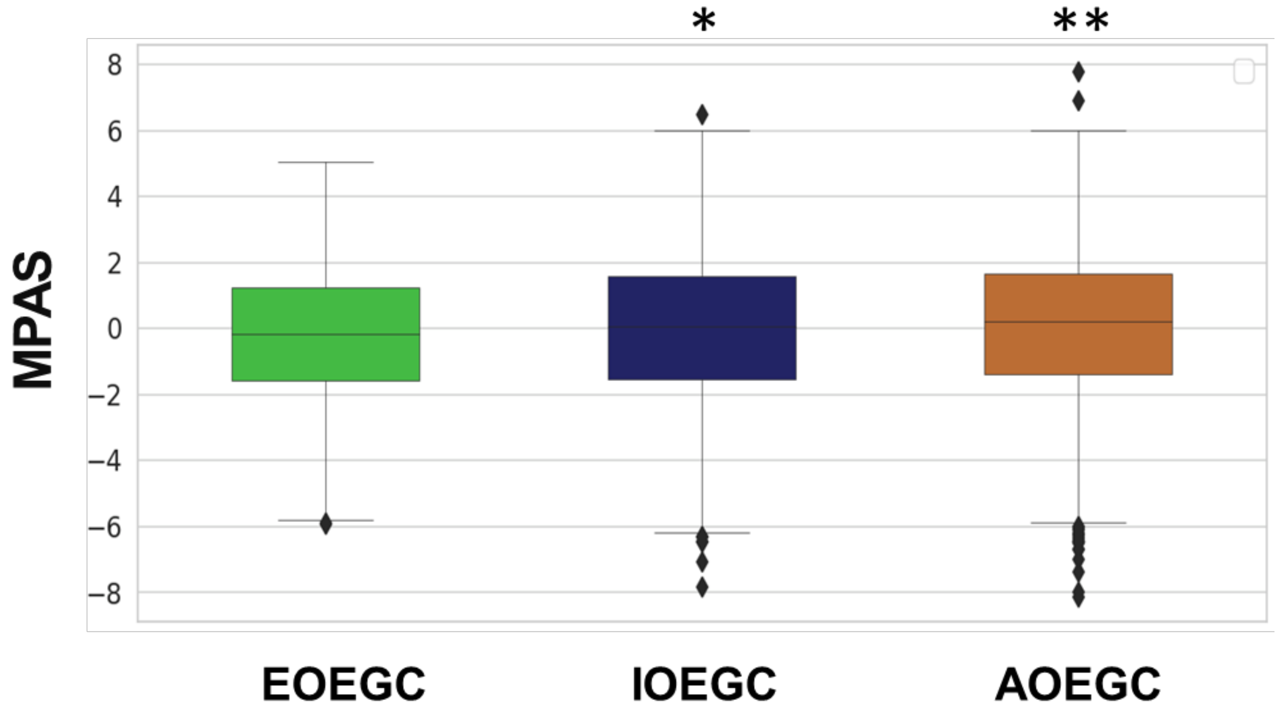

Supplementary Figure 3: Real world overall survival (rwOS) for patients with EOEGC, IOEGC, and AOEGC stratified by treatment. (A) Kaplan-Meier curves of rwOS of patients stratified by dMMR/MSI-H status with EOEGC, IOEGC, AOEGC after treatment with immune checkpoint inhibitor pembrolizumab or nivolumab. (B) rwOS stratified by TMB-High status after treatment with immune checkpoint inhibitor. (C) rwOS stratified by treatment with trastuzumab for patients with HER2-positive tumors. Log-rank test was performed with significance determined as $p < .05$.

A

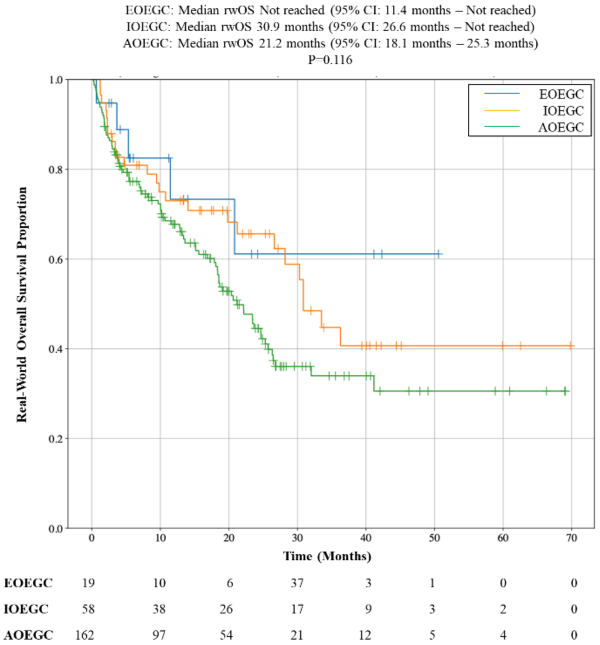

B

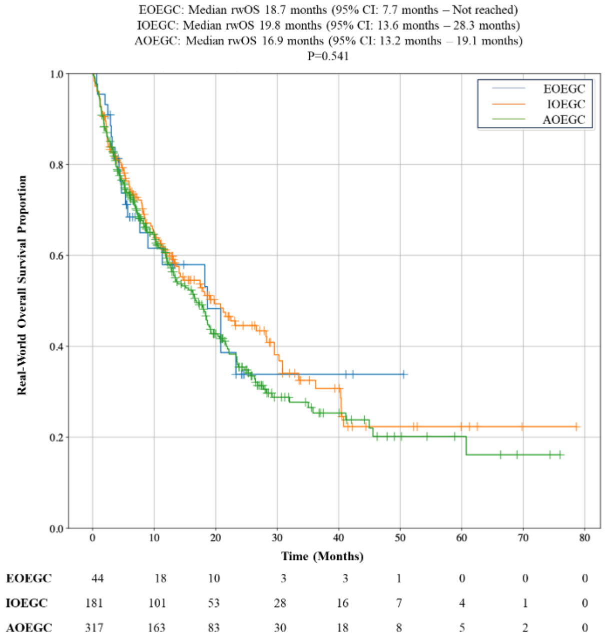

C

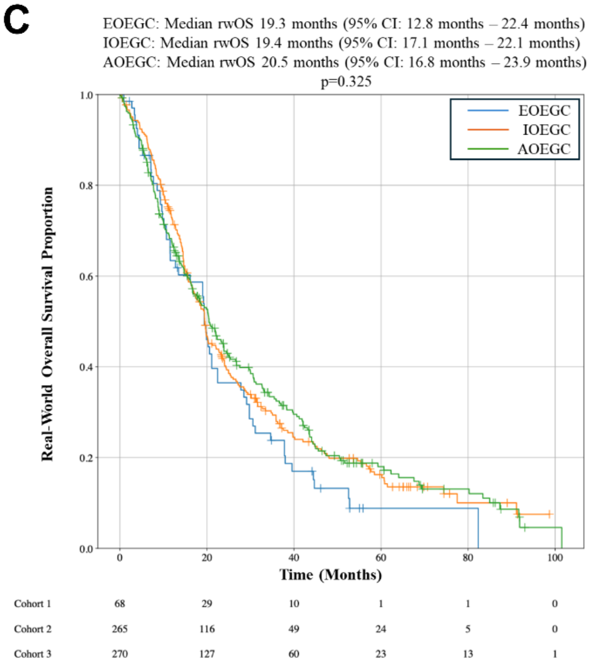

Supplementary Table 1: Mutation analysis

Mutation		EOEGC	IOEGC	AOEGC
CDH1	%	18.88	7.15	6.09
	% Change		11.74	12.79
	p-value		1.1E-14	2.9E-22
	q-value		8.3E-12	4.4E-19
TP53	%	65.90	74.51	74.40
	% Change		-8.60	-8.50
	p-value		0.0001	5.7E-05
	q-value		0.006	0.004
ARID2	%	0.58	2.89	4.14
	% Change		-2.31	-3.56
	p-value		0.002	6.5E-05
	q-value		0.07	0.004
APC	%	3.47	6.36	8.45
	% Change		-2.89	-4.98
	p-value		0.01	9.4E-05
	q-value		0.25	0.005
KMT2D	%	4.61	6.23	9.89
	% Change		-1.62	-5.28
	p-value		0.18	0.0002
	q-value		0.98	0.008
RNF43	%	1.93	3.01	5.38
	% Change		-1.08	-3.44
	p-value		0.19	0.0008
	q-value		1.00	0.03
KMT2A	%	0.39	1.69	2.83
	% Change		-1.31	-2.45
	p-value		0.03	0.001
	q-value		0.38	0.04
CDKN2A	%	8.84	12.37	14.25
	% Change		-3.53	-5.41
	p-value		0.03	0.001
	q-value		0.40	0.04
HNF1A	%	0.39	1.39	2.72
	% Change		-1.00	-2.34
	p-value		0.06	0.001
	q-value		0.65	0.05
ASXL1	%	1.14	1.80	4.70
	% Change		-0.66	-3.55
	p-value		0.40	0.002
	q-value		1.00	0.07
NOTCH1	%	0.70	1.90	3.24
	% Change		-1.19	-2.54
	p-value		0.09	0.004
	q-value		0.76	0.11
ARID1A	%	13.74	13.57	18.94
	% Change		0.17	-5.20

	p-value		0.92	0.005
	q-value		1.00	0.12
KDM6A	%	0.60	2.39	2.70
	% Change		-1.79	-2.10
	p-value		0.01	0.005
	q-value		0.25	0.12
SMAD4	%	5.00	8.17	8.19
	% Change		-3.17	-3.19
	p-value		0.02	0.01
	q-value		0.29	0.25
CREBBP	%	0.78	1.26	2.57
	% Change		-0.48	-1.79
	p-value		0.37	0.01
	q-value		1.00	0.25
SMARCA4	%	2.54	3.63	5.01
	% Change		-1.09	-2.47
	p-value		0.23	0.01
	q-value		1.00	0.28
ATM	%	1.72	2.86	3.86
	% Change		-1.14	-2.14
	p-value		0.15	0.02
	q-value		0.94	0.28
CTNNB1	%	1.35	3.02	3.32
	% Change		-1.66	-1.97
	p-value		0.04	0.02
	q-value		0.48	0.29
GNAS	%	0.97	1.20	2.73
	% Change		-0.23	-1.76
	p-value		0.67	0.02
	q-value		1.00	0.30
KMT2C	%	2.29	2.64	4.43
	% Change		-0.35	-2.15
	p-value		0.67	0.03
	q-value		1.00	0.40
MED12	%	3.96	1.00	0.85
	% Change		2.96	3.11
	p-value		0.07	0.04
	q-value		0.68	0.46

Comparative analysis of molecular alterations between cohorts calculated with chi-square or Fisher’s exact test. P and Q values calculated from statistical comparison of cohort relative to EOEGC.

Supplementary Table 2: Copy number alteration analysis

Copy Number Alteration		EOEGC	IOEGC	AOEGC
CCND1	%	2.70	9.39	8.64
	% Change		-6.69	-5.93
	p-value		7.0E-07	3.3E-06
	q-value		0.0001	0.0003
FGF19	%	2.91	9.53	8.92
	% Change		-6.62	-6.01
	p-value		1.2E-06	3.8E-06
	q-value		0.0002	0.0003
FGF4	%	3.32	9.00	8.39
	% Change		-5.68	-5.07
	p-value		2.3E-05	7.0E-05
	q-value		0.002	0.004
FGF3	%	4.01	9.69	9.17
	% Change		-5.68	-5.16
	p-value		5.9E-05	0.0001
	q-value		0.004	0.006
CCNE1	%	6.81	5.36	3.79
	% Change		1.45	3.02
	p-value		0.22	0.002
	q-value		1.00	0.06
FGFR2	%	3.45	2.47	1.52
	% Change		0.98	1.93
	p-value		0.23	0.002
	q-value		1.00	0.07
EGFR	%	2.87	3.99	5.27
	% Change		-1.12	-2.40
	p-value		0.24	0.02
	q-value		1.00	0.31
MYC	%	6.13	4.52	3.91
	% Change		1.61	2.22
	p-value		0.14	0.02
	q-value		0.94	0.33
HSP90AB1	%	0.38	1.00	1.27
	% Change		-0.62	-0.88
	p-value		0.28	0.08
	q-value		1.00	0.72
CRTC3	%	0.58	0.23	0.14
	% Change		0.34	0.44
	p-value		0.21	0.08
	q-value		1.00	0.72
AKT2	%	0.97	0.29	0.39
	% Change		0.67	0.58
	p-value		0.06	0.09
	q-value		0.63	0.76

Comparative analysis of molecular alterations between cohorts calculated with chi-square or Fisher’s exact test. P and Q values calculated from statistical comparison of cohort relative to EOEGC.

Supplementary Table 3: Fusion analysis

Fusion		EOEGC	IOEGC	AOEGC
ARHGAP26	%	5.67	3.68	1.31
	% Change		2.00	4.36
	p-value		0.04	3.7E-11
	q-value		0.53	1.9E-08
ROS1	%	0.38	0.06	0.03
	% Change		0.32	0.34
	p-value		0.14	0.06
	q-value		0.94	0.65
CLDN18	%	0.38	0.23	0.03
	% Change		0.15	0.34
	p-value		0.63	0.06
	q-value		1.00	0.65

Comparative analysis of molecular alterations between cohorts calculated with chi-square or Fisher’s exact test. P and Q values calculated from statistical comparison of cohort relative to EOEGC.

Supplementary Table 4: ARHGAP26 fusion components

Fusion	N
CLDN18-ARHGAP26	42
CTNND1-ARHGAP26	9
OCLN-ARHGAP26	8
ARHGAP26-KIAA0141	2
ARHGAP26-LOC101926941	2
ARHGAP26-NR3C1	2
ARHGAP26-CCDC155	1
ARHGAP26-DOCK2	1
ARHGAP26-GALNT10	1
ARHGAP26-KCTD16	1
ARHGAP26-MAGI1	1
ARHGAP26-SIMC1	1
ARHGAP26-SOX2-OT	1
ARHGAP26-STK32A	1
ARHGAP26-WLS	1
NR3C1-ARHGAP26	1

Supplementary Table 5: HER2 expression and amplification analysis

		EOEGC	IOEGC	AOEGC
RNA-Her2 (TPM)	Median	4.92	4.93	4.92
	p-value		0.62	0.70
	q-value		0.79	0.79
IHC-Her2	% (N Pos)	7.79 (36)	9.57 (146)	9.50 (240)
	p-value		0.25	0.24
	q-value		1.00	1.00
CNA-Her2	% (N Pos)	7.9 (41)	10.58 (180)	8.81 (250)
	p-value		0.07	0.50
	q-value		0.71	1.00
CISH-Her2	% (N Pos)	13.33 (38)	19.5 (175)	16.5 (238)
	p-value		0.02	0.18
	q-value		0.30	0.98

Comparative analysis of molecular alterations between cohorts calculated with chi-square or Fisher's exact test. P and Q values calculated from statistical comparison of cohort relative to EOEGC.

Supplementary Table 6: Immune-oncology markers

		EOEGC	IOEGC	AOEGC
TMB High	% (N Pos)	3.84 (20)	7.82 (135)	11.52 (329)
	p-value		0.002	1.2E-07
	q-value		0.05	3.1E-05
dMMR/MSI-H	% (N Pos)	1.32 (7)	3.34 (58)	7.05 (204)
	p-value		0.02	4.7E-07
	q-value		0.28	9.2E-05
IHC-PD-L1 (22c3)	% (N Pos)	68.12 (329)	70.46 (1107)	73.18 (1943)
	p-value		0.32	0.02
	q-value		1.00	0.34

Comparative analysis of molecular alterations between cohorts calculated with chi-square or Fisher's exact test. P and Q values calculated from statistical comparison of cohort relative to EOEGC.

Supplementary Table 7: Immune cell infiltrate

	Median			p-value		q-value	
	EOEGC	IOEGC	AOEGC	EOEGC vs IOEGC	EOEGC vs AOEGC	EOEGC vs IOEGC	EOEGC vs AOEGC
B cells	0.047	0.040	0.039	2.0E-08	4.5E-11	1.6E-07	1.5E-09
Mφ M1	0.037	0.041	0.040	0.003	0.002	0.007	0.006
Mφ M2	0.036	0.031	0.030	7.7E-07	3.2E-10	5.1E-06	5.3E-09
Monocytes	0.0	0.0	0.0	0.02	0.0004	0.04	0.002
Neutrophils	0.054	0.059	0.058	0.33	0.42	0.47	0.56
Natural killer cells	0.028	0.025	0.025	3.4E-06	7.0E-09	1.8E-05	7.7E-08
T cells CD4	0.0	0.0	0.0	0.45	0.01	0.57	0.03
T cells CD8	0.001	0.0005	0.001	0.58	0.84	0.68	0.86
T regulatory cells	0.022	0.022	0.023	0.72	0.05	0.77	0.10
Dendritic cells	0.005	0.003	0.003	0.001	0.001	0.004	0.004

Computationally inferred intratumoral immune population with the median immune fraction according to quanTIseq. Tumor microenvironment cell fractions were analyzed among cohorts using nonparametric Kruskal-Wallis testing.

Supplementary Table 8: Immune checkpoint gene expression

		EOEGC	IOEGC	AOEGC
CD274	Median	2.32	2.48	2.54
	p-value		0.04	8.2E-05
	q-value		0.10	0.002
FOXP3	Median	1.76	1.81	1.87
	p-value		0.02	0.002
	q-value		0.07	0.02
HAVCR2	Median	3.84	3.74	3.72
	p-value		0.02	0.02
	q-value		0.07	0.07
LAG3	Median	0.84	0.86	0.88
	p-value		0.33	0.07
	q-value		0.47	0.13
PDCD1	Median	0.58	0.55	0.55
	p-value		0.40	0.18
	q-value		0.50	0.29
PDCD1LG2	Median	0.93	0.94	0.97
	p-value		0.69	0.10
	q-value		0.76	0.19
CTLA4	Median	1.51	1.51	1.54
	p-value		0.35	0.06
	q-value		0.47	0.13
IDO1	Median	1.75	1.70	1.83
	p-value		0.92	0.03
	q-value		0.94	0.10

Fold change gene expression levels in transcripts per million (TPM) of immune checkpoint genes IOEGC relative to EOEGC or AOEGC relative to EOEGC. Mann-Whitney U test used to determine statistically significant differences in immune gene expression.

Supplementary Table 9: Gene Set Enrichment Analysis for EOEGC versus IOEGC

Pathway	NES	FDR
COAGULATION	1.83	0.00
EMT	1.60	0.02
E2F TARGETS	-1.74	0.02
G2M CHECKPOINT	-1.69	0.03
MTORC1 SIGNALING	-1.59	0.03
MYC TARGETS V2	-1.49	0.09
MYC TARGETS V1	-1.34	0.27
MYOGENESIS	1.35	0.37
KRAS SIGNALING DN	1.24	0.53
SPERMATOGENESIS	-1.23	0.59
ANGIOGENESIS	1.25	0.60
HEDGEHOG SIGNALING	1.16	0.62
BILE ACID METABOLISM	1.26	0.63

UV RESPONSE DN	1.17	0.63
PANCREAS BETA CELLS	1.14	0.63
TNFA SIGNALING VIA NFKB	-1.20	0.65
NOTCH SIGNALING	1.17	0.70
KRAS SIGNALING UP	1.19	0.71
GLYCOLYSIS	-1.15	0.72
UNFOLDED PROTEIN RESPONSE	-1.16	0.77
APICAL JUNCTION	1.08	0.83
ESTROGEN RESPONSE LATE	-0.99	0.88
PI3K AKT MTOR SIGNALING	-1.00	0.89
HYPOXIA	-1.10	0.90
DNA REPAIR	-1.07	0.93
MITOTIC SPINDLE	-1.04	0.94
COMPLEMENT	0.98	0.94
CHOLESTEROL HOMEOSTASIS	-1.00	0.95
HEME METABOLISM	-0.64	0.98
UV RESPONSE UP	-0.91	0.98
INTERFERON GAMMA RESPONSE	-0.55	0.98
APICAL SURFACE	-1.01	0.99
TGF BETA SIGNALING	0.54	0.99
ALLOGRAFT REJECTION	0.99	0.99
XENOBIOTIC METABOLISM	0.99	1.00
ADIPOGENESIS	0.89	1.00
WNT BETA CATENIN SIGNALING	0.82	1.00
PEROXISOME	0.87	1.00
INFLAMMATORY RESPONSE	-0.71	1.00
PROTEIN SECRETION	-0.82	1.00
REACTIVE OXYGEN SPECIES PATHWAY	-0.72	1.00
IL2 STAT5 SIGNALING	0.81	1.00
FATTY ACID METABOLISM	0.78	1.00
OXIDATIVE PHOSPHORYLATION	-0.64	1.00
ANDROGEN RESPONSE	-0.70	1.00
ESTROGEN RESPONSE EARLY	0.73	1.00
APOPTOSIS	0.68	1.00
P53 PATHWAY	-0.72	1.00
INTERFERON ALPHA RESPONSE	0.55	1.00
IL6 JAK STAT3 SIGNALING	0.58	1.00

Gene set enrichment analysis (GSEA) differences in pathways based on normalized enrichment scores (NES) in EOEGC versus IOEGC. Positive NES would imply higher values in EOEGC. False discovery rate (FDR) < 0.25 considered statistically significant.

Supplementary Table 10: Gene Set Enrichment Analysis for EOEGC versus AOEGC

Pathway	NES	FDR
EMT	1.82	0.00
G2M CHECKPOINT	-1.66	0.03
MYC TARGETS V2	-1.55	0.03
MYOGENESIS	1.66	0.04
COAGULATION	1.64	0.04
MTORC1 SIGNALING	-1.59	0.04
E2F TARGETS	-1.71	0.07
MYC TARGETS V1	-1.41	0.16
UNFOLDED PROTEIN RESPONSE	-1.39	0.17
CHOLESTEROL HOMEOSTASIS	-1.38	0.18
ANGIOGENESIS	1.41	0.26
UV RESPONSE DN	1.31	0.46
DNA REPAIR	-1.24	0.57
BILE ACID METABOLISM	1.25	0.57
HEDGEHOG SIGNALING	1.20	0.62
KRAS SIGNALING DN	1.16	0.68
KRAS SIGNALING UP	1.21	0.68
APICAL JUNCTION	1.12	0.73
ESTROGEN RESPONSE LATE	-1.10	0.74
NOTCH SIGNALING	0.99	0.76
APICAL SURFACE	-1.11	0.76
COMPLEMENT	1.08	0.77
ALLOGRAFT REJECTION	1.01	0.77
PI3K AKT MTOR SIGNALING	-1.11	0.81
PANCREAS BETA CELLS	1.02	0.82
UV RESPONSE UP	-1.04	0.85
GLYCOLYSIS	-1.12	0.85
XENOBIOTIC METABOLISM	1.03	0.86
TNFA SIGNALING VIA NFKB	-1.14	0.87
FATTY ACID METABOLISM	0.68	0.89
HEME METABOLISM	-0.70	0.89
SPERMATOGENESIS	-1.16	0.90
PROTEIN SECRETION	-0.98	0.91
IL6 JAK STAT3 SIGNALING	0.69	0.92
INFLAMMATORY RESPONSE	-0.73	0.93
IL2 STAT5 SIGNALING	0.86	0.93
APOPTOSIS	-0.64	0.93
ESTROGEN RESPONSE EARLY	-0.70	0.93
MITOTIC SPINDLE	-0.99	0.94
INTERFERON GAMMA RESPONSE	-0.75	0.94
TGF BETA SIGNALING	0.74	0.95
PEROXISOME	0.70	0.96
P53 PATHWAY	-0.93	0.96
ADIPOGENESIS	0.80	0.97
ANDROGEN RESPONSE	-0.76	0.98

WNT BETA CATENIN SIGNALING	0.77	0.98
REACTIVE OXYGEN SPECIES PATHWAY	-0.83	1.00
INTERFERON ALPHA RESPONSE	-0.77	1.00
HYPOXIA	-0.81	1.00
OXIDATIVE PHOSPHORYLATION	-0.81	1.00

Gene set enrichment analysis (GSEA) differences in pathways based on normalized enrichment scores (NES) in EOEGC versus AOEGC. Positive NES would imply higher values in EOEGC. False discovery rate (FDR) < 0.25 considered statistically significant.

Supplementary Table 11: MAPK Pathway Activity Score (MPAS) analysis

		EOEGC	IOEGC	AOEGC
MPAS	Median	-0.17	0.04	0.18
	p-value		0.04	0.001
	q-value		0.06	0.004

Mann-Whitney U test was used to compare cohorts to EOEGC.
